# The association of hypogonadism with depression and its treatments

**DOI:** 10.3389/fendo.2023.1198437

**Published:** 2023-08-10

**Authors:** Rita Indirli, Valeria Lanzi, Maura Arosio, Giovanna Mantovani, Emanuele Ferrante

**Affiliations:** ^1^ Endocrinology Unit, Fondazione IRCCS Ca’ Granda Ospedale Maggiore Policlinico, Milan, Italy; ^2^ Department of Clinical Sciences and Community Health, University of Milan, Milan, Italy

**Keywords:** depression, male hypogonadism, testosterone, testosterone replacement therapy (TRT), antidepressants

## Abstract

According to World Health Organization estimates, 5% of the adult population worldwide suffers from depression. In addition to the affective, psychomotor and cognitive symptoms which characterize this mood disorder, sexual dysfunction has been frequently reported among men suffering from depression. The most common sexual manifestations are decreased libido, erectile dysfunction and orgasmic disorder. In addition, epidemiological studies have documented a reduction of testosterone concentrations in men with depression and, for these reasons, depressive disorders appear as one possible cause of male functional hypogonadism. Moreover, some largely used antidepressant medications can cause or worsen sexual complaints, thus depression and its treatments rise several andrological-relevant issues. The other way round, men with hypogonadism can manifest depressed mood, anxiety, insomnia, memory impairment which, if mild, may respond to testosterone replacement therapy (TRT). However, the prevalence of functional hypogonadism in depression, and of depressive symptoms in hypogonadal men, is not known. Severe depressive symptoms do not respond to TRT, while the effect of treating major depression on functional hypogonadism, has not been investigated. Overall, the clinical relevance of each condition to the other, as well as the physiopathological underpinnings of their relationship, are still to be clarified. The present review summarizes current evidence on the influence of testosterone on mood and of depression on the hypothalamic-pituitary-testis axis; the clinical association between male hypogonadism and depression; and the reciprocal effects of respective treatments.

## 1 Introduction

Male hypogonadism is a clinical and biochemical syndrome associated with low levels of testosterone (1–3). While the most specific symptoms are the sexual ones, i.e. low libido, erectile dysfunction, diminished frequency of morning erections (4) and orgasmic disorders (5), other non-specific manifestations are included in the syndrome, like fatigue, cognitive impairment and depressed mood (1, 3).

Depressive symptoms have been reported in 35-50% of male patients with hypogonadism in cross-sectional studies (6–8). Hypogonadism at baseline has been associated with development of depressive disorders in some short (9) and long term longitudinal studies (10), even if negative results have been reported as well (11). With the aim of characterizing their emotional state, Lašaite and colleagues compared the scores obtained in questionnaires on mood, quality of life and cognitive functioning by 34 young hypogonadal men and 34 age-matched healthy controls (12). In this way, authors identified higher levels of depression, fatigue, confusion and inactivity among patients with hypogonadism compared to healthy controls. Interestingly, the social and psychological domains of quality of life and some cognitive functions were impaired too. Likewise, young patients affected with congenital hypogonadotropic hypogonadism, displayed difficulty in emotional role, reduced vitality and a higher prevalence of depression compared to age-matched controls, as assessed by Short Form 36 and the Beck Depression Inventory (13). Overall, evidence suggests that low testosterone may cause depressed mood in at least a subset of hypogonadal men.

The other way round, men with depressive disorders can exhibit features consistent with hypogonadism. According to the latest edition of the Diagnostic and Statistical Manual of Mental Disorders (DSM-5), depressive disorders are defined by depressed mood, loss of interest or pleasure in most activities, and a range of symptoms including psychomotor changes with agitation or retardation, low energy, impaired ability to concentrate or make decisions (14). However, besides the affective, psychomotor and cognitive symptoms, depression can bring about vegetative manifestations which encompass unintentional weight loss or gain, insomnia or hypersomnia, and sexual dysfunction.

A recent meta-analysis has reported that sexual dysfunction can be observed in 63% of men with major depressive disorder overall; it manifests with decreased libido in 40% of male patients; erectile dysfunction in 32%; and orgasmic disorder in 35% (15). Furthermore, a systematic review with meta-analysis of case-control studies on male depressive disorder, has reported that patients have significantly lower plasma testosterone concentrations than healthy controls (16). Moreover, depression severity is significantly and inversely associated with levels of bioavailable and free testosterone and dihydrotestosterone (8, 17, 18). Therefore, depression should be considered a comorbid illness leading to a condition of *functional hypogonadism*, i.e. hypogonadism due to potentially reversible disruption of the hypothalamic–pituitary–testis (HPT) axis functioning (1).

Some epidemiologic clues further suggest association between sex steroids and mood. Depression shows different prevalence and features between men and women: the prevalence of affective disorders in women is twice as high as in men (19), and male depressed patients display lower stress tolerance, worse impulse control, a higher prevalence of antisocial behaviour and suicide compared to female subjects (19). Male hypogonadism and depression are age-related, as both conditions have a higher prevalence in older men (20, 21) and may have a reciprocal influence: if, on one hand, sexual issues can result in depression and social withdrawal, on the other hand sexual thinking is affected by psychological disturbances, including depression, in older men (22).

The aim of this review is to explore the physiopathology behind the influence of testosterone on mood and of depression on the HPT axis; to examine the clinical association between male hypogonadism and depression; and to summarize evidence on the effects of testosterone therapy on depression, and of antidepressants on male sexual function.

## 2 Methods

A primary literature search was performed in PubMed including the following keywords: “male hypogonadism”, “testosterone”, “sexual symptoms”, “sexual dysfunction”, “depression”, “mood”, using the Boolean functions AND/OR. The search was restricted to English-language studies published up to December 2022. The reference lists of the identified papers were also scrutinized for further pertinent studies.

Narrative reviews, systematic reviews, human observational studies, clinical trials and animal studies were included. If it was not clear from the abstract whether the study contained relevant data, the full text was retrieved. The eligibility criteria for selection were as follows: 1) animal studies investigating the behavioural response to HPT hormones, and/or their expression and effects in cerebral areas related to mood disorders; 2) human studies investigating the relationship between endogenous HPT hormones’ levels, androgen therapy, sexual dysfunction, depressive symptoms, antidepressant medications, and activity of cerebral areas related to mood disorders as assessed by morphologic or functional studies.

## 3 The reciprocal influence of HPT axis and depression

### 3.1 Effects of depression on the HPT axis

#### 3.1.1 The HPT axis

An in-depth description of the HPT axis functioning and regulation is beyond the purpose of this review. Shortly, the preoptic area and the infundibular nucleus of the hypothalamus contain neurons producing gonadotropin releasing hormone (GnRH) (23). They project to the median eminence, so that GnRH is secreted into portal circulation to reach the anterior pituitary. GnRH is released in a pulsatile pattern and determines the pulsatile secretion of gonadotropins, luteinizing hormone (LH) and follicle stimulating hormone (FSH), into systemic circulation (23). Finally, LH acts on testis where it stimulates Leydig cells to produce testosterone (24).

Even though GnRH neurons have intrinsic electrical pulsatility, their activity is synchronized by an external hypothalamic *pulse generator* which encompasses the kisspeptin-neurokinin B-dynorphin pathway mainly, but also glutamatergic cells and other neurons (23). Kisspeptin neurons are located in the preoptic area and the infundibular nucleus of the hypothalamus. In the latter, Kisspeptin neurons co-express neurokinin B and dynorphin and are called KNDy neurons. Through the (stimulatory) neurokinin B receptor and the (inhibitory) kappa opioid peptide receptor, KNDy neurons regulate kisspeptin secretion in an autologous fashion (23).

Kisspeptin is the most potent GnRH secretagogue currently known. However, other mediators influence the GnRH-gonadotrope axis, including gamma-amino butyric acid (GABA), vasoactive intestinal polypeptide, vasopressin, catecholamines, nitric oxide, neurotensin, gonadotropin-inhibitory hormone (GnIH)/RFamide related peptide-3 (RFRP-3) (23). In mammals, RFRP-3 neurons have been identified in the hypothalamic dorsomedial nucleus, and they act by inhibiting GnRH- and kisspeptin-secreting cells. Through these complex interactions, a multitude of stimuli can modulate the HPT axis, including stress, inflammation, opioid drugs (23).

#### 3.1.2 Functional hypogonadism in depressive disorders: physiopathological hypotheses

As stated above, depressive disorders can be associated with functional hypogonadotropic hypogonadism. Many aspects of depression may contribute to this outcome, including weight loss, disrupted sleep, hyperactivity of the hypothalamus-pituitary-adrenal axis, and psychomotor retardation.

In some patients, depression entails loss of appetite which results in inadequate energy intake and weight loss. In this setting, HPT axis dysfunction stems at least in part from reduction in circulating levels of leptin (25). Leptin is an anorexigenic adipokine, the levels of which drop in response to starvation. It acts in different brain regions including the hypothalamus (26), where 40% of Kisspeptin neurons express leptin receptor (23). The loss of GnRH pulsatility in food deprivation results from the lack of leptin stimulation on kisspeptin neurons (25).

Sleep disorders, i.e. difficulty initiating or maintaining sleep, with or without hypersomnolence, are common in depression, where insomnia prevalence exceeds 80% (27). Moreover, longitudinal studies suggest that insomnia is a risk factor for depression, as it is associated with depressive symptom severity, lower rates of remission, higher risk of relapse, suicidal thoughts and self-injury (28). On the other hand, sleep disturbances can impact testosterone concentrations and sexual symptoms. In a study on shift workers, authors distinguished participants who suffered from shift work sleep disorder, i.e. a circadian rhythm disorder characterized by excessive day-time sleepiness, from participants who did not (29). The former were found to have worse hypogonadal symptoms and lower testosterone levels than shift workers without shift work sleep disorder and day-time workers. Authors conclude that poor sleep habits may contribute to hypogonadal symptoms severity. In another study analysing association between sex hormones concentration and self-rated health and life satisfaction in elderly men, lower free testosterone concentrations were significantly associated with sleeping problems (30). Among 48 male patients treated at a men’s health clinic for andrological problems, 32 (67%) were classified as poor sleepers according to the Pittsburgh Sleep Quality Index (31).

Serum cortisol concentrations are significantly increased in patients with depressive disorder, as a result of hypothalamus-pituitary-adrenal axis hyperactivity (32). Cortisol but also upstream factors like corticotropin releasing hormone and vasopressin, exert negative effects at both pituitary and hypothalamic levels (23). Cortisol treatment reduced pulse frequency of GnRH measured in ovine pituitary portal blood (33). Both the hypothalamic corticotropin releasing hormone and corticosterone downregulated the expression of Kisspeptin gene in rats (34). Administration of hydrocortisone to eumenorrheic women reduced LH pulse frequency, an outcome which most likely resulted from interference with GnRH pulsatile release (35). Male patients with endogenous hypercortisolism present hypogonadotropic hypogonadism, which generally resolves upon hypercortisolism remission (36). Moreover, in patients affected with Cushing’s syndrome, a blunted response of gonadotropins to exogenous GnRH administration has been documented, a finding which points to a direct inhibitory effect of excess cortisol at the pituitary level (36, 37).

Psychomotor retardation is a central, complex feature of depressive disorders which encompasses modifications in individual motility, mental activity, and speech (38). As far as motility is concerned, psychomotor retardation impairs gross and fine motor activity, eye movements, facial movements (38). Together with other symptoms of depression like loss of interest in most activities and low energy, psychomotor retardation contributes to physical inactivity in depressed subjects (39). Actually, evidence on the effects of physical exercise on testosterone levels is controversial (40). While some studies show that testosterone levels rise acutely after training, others support a long-term effect; however, reduction and no variation of testosterone levels have been reported in relation to physical exercise as well (40). This variability can be ascribed to numerous factors which influence the relationship between physical activity and testosterone production, like the type of exercise (endurance or resistance), training intensity and duration, subjects’ features (age, body weight, sedentary lifestyle) (40).

Interestingly, somatization has been suggested as a factor potentially determining or exacerbating sexual symptoms (41). In a retrospective study analysing 2833 patients complaining sexual symptoms, Fanni et al. observed that patients scoring higher on a “somatized anxiety symptoms” scale, were older and more obese, and reported unhealthy lifestyle and a lower education, along with sexual impairment, with a higher frequency. A significant association between the “somatized anxiety symptoms” score and low testosterone levels was observed too. Therefore, we suggest that somatization could be one more mechanism underlying sexual dysfunction in depressed patients.

Finally, neuronal circuits involved in the physiopathology of mood disorders, may be directly responsible for HPT axis deregulation. However, to the best of our knowledge, data on this intriguing hypothesis are not currently available.

### 3.2 Effects of HPT hormones on mood modulation

Hormones of the HPT axis, i.e. Kisspeptin, GnIH/RFRP-3, GnRH, gonadotropins, testosterone and estradiol, act on cerebral areas involved in the pathogenesis of mood disorders, like the hippocampus and the limbic system, particularly the amygdala (42, 43).

#### 3.2.1 Action of HPT hormones in central nervous system

Neuronal fibers containing kisspeptin and GnIH/RFRP are found not only in the hypothalamus, but also in amygdala, hippocampus, habenula, periaqueductal gray, and ventral tegmental area in mammals (44). Consistently, Kisspeptin receptors expression has been documented in several regions of human brain, which include hippocampus and amygdala (45).

The dysfunction in serotoninergic neurotransmission has been implicated in mood disorders (46). In zebrafish, kisspeptin has been found to interact with the serotonergic system during a substance-evoked alarm experiment (47). Administration of kisspeptin in male mice during a forced swimming test resulted in antidepressive-like effects, which were at least in part mediated by the interaction with α_2_-adrenergic and 5-hydroxytryptamine-type 2 (5-HT_2_) serotonergic receptors (48).

In rodents, administration of GnRH agonist exerts antidepressant effect, and anxiolytic effect comparable to diazepam, whereas GnRH antagonist increases anxiety levels (49, 50). These observations were confirmed in castrated animals, ruling out the involvement of peripheral sex hormones in the anxiolytic and antidepressant actions of GnRH.

Animal studies have demonstrated that the preoptic area, the hypothalamus and the amygdala are targets of potent, non-aromatizable androgens (51, 52). Additionally, androgens influence dopaminergic neurotransmission to the caudate putamen, nucleus accumbens and amygdala in rats (53). Apostolinas and colleagues showed that androgen receptor expression is found in medial amygdala, preoptic area, lateral ventral septum, and stria terminalis, and is modulated by testosterone levels (54). Moreover, testosterone may exert neuroprotective effects: indeed, Sarchielli and colleagues showed that testosterone administration reduced high-fat diet induced hypothalamic inflammation in an animal model of metabolic syndrome (55).

The conversion of testosterone by the enzyme aromatase results in the production of estrogen, which acts in target tissues through estrogen receptors α and β. Biegon et al. studied aromatase distribution in the healthy human brain (56): distribution volume values were measured using positron emission tomography (PET) with the radiolabeled aromatase inhibitor [N-methyl-^11^C] vorozole. High aromatase expression was observed in the thalamus and the amygdala. Using *in situ* hybridization histochemistry on post-mortem specimens, Osterlund and colleagues identified human cerebral areas containing ribonucleic acid (mRNA) of estrogen receptors (57). The highest expression of estrogen receptor α was found in the amygdala and hypothalamus, while that of estrogen receptor β in the temporal cortex, claustrum, thalamus and hippocampus. In particular, estrogen has been shown to act on serotonergic neurons in rats (58) and primates (59) and reduce anxiety- and depression-like behaviors in females (60). Therefore, testosterone may be involved in mood disorders’ physiopathology through the conversion in estrogen and the subsequent action on estrogen receptors in the limbic system.

It has to be mentioned that the nervous system not only responds to sex steroids released by testicles into circulation, but it has its own steroidogenic activity which leads to production of testosterone and estrogen as well (61). Neurosteroids activate the classical nuclear receptors (androgen receptor and estrogen receptors α and β), but some of them may act through different pathways. For instance, estradiol also binds the membrane G protein coupled estrogen receptor (GPER) (62), while other steroid metabolites may activate non-classical steroid receptors, including dopamine 1 receptor, GABA receptors A and B, serotonin type 3 receptors, both at synaptic and extra-synaptic sites (61). To which extent neurosteroids interact with peripherally synthetized sex steroids to influence mood, is still to be clarified.

#### 3.2.2 Morphologic studies

Voxel based morphometry has been used to study the relationship between testosterone levels and grey and white matter volumes in humans. A significantly positive correlation has been identified for grey matter volume in the hippocampus, amygdala, hypothalamus and mammilary bodies (63, 64). Animal studies have shown that androgen deprivation causes a reduction in synaptic density in the hippocampus, which recovers after testosterone replacement (65).

#### 3.2.3 Functional studies

Functional studies using functional magnetic resonance imaging (fMRI) or PET have found an association between testosterone levels and activation of distinct brain regions (66–68). In a study on young boys affected with familial male precocious puberty, patients with early excess testosterone secretion displayed more intense activation of the hippocampus when shown fearful faces compared to age-matched healthy controls (69). Derntl et al. performed fMRI in 21 healthy men during an emotion recognition protocol to detect amygdala activation (70). A positive correlation was found between testosterone concentrations and amygdala response to fearful and angry facial expressions. Hence, authors conclude that testosterone affects threat-related amygdala activation. In another study employing fMRI to assess amygdala activation in response to threat-related stimuli, Manuck et al. reported that reactivity in the ventral amygdala of adult men was inversely correlated with number of CAG repeats in the androgen receptor-encoding gene; conversely, activation of dorsal amygdala correlated positively with salivary testosterone, but not with number of CAG repeats (71). The latter finding suggests that testosterone effects in dorsal amygdala may even be mediated by non-genomic or androgen receptor-independent pathways.

Comninos et al. used a combination of functional studies and psychometric analyses to assess effects of kisspeptin in 29 healthy young men (72). Kisspeptin administration enhanced brain activity in limbic areas in response to sexual stimuli, and this finding correlated with psychometric measures of reward, drive, sexual aversion and mood, in particular with attenuation of negative mood. When participants were shown negative-evoked visual stimuli like images of car crashes or terminal patients, kisspeptin enhanced activity of prefrontal cortex (72), which has a role in reducing fear and anxiety in response to negative stimuli (73).

### 3.3 Conclusions

Many clues suggest that HPT axis hormones, neurotransmission and cerebral areas involved in mood disorders, can influence each other. Depression symptoms and physiopathological aspects like weight loss, sleep disorders, activation of the hypothalamus-pituitary-adrenal axis, and physical inactivity can lead to functional hypogonadism.

The other way round, testosterone and other hormones of the HPT axis can act on a multitude of cerebral areas and modulate neurotransmission, including the ones involved in mood disorders. Indeed, neuro-hormonal fibers, hormone receptors and metabolic enzymes are largely distributed in the central nervous system. Morphologic and functional studies have confirmed the effects of sexual hormones in cerebral regions of interest. **Table 1** summarizes current evidence on these aspects.

**Table 1:** Summary of current evidence on the effects of hypothalamus-pituitary-testis axis hormones in central nervous system.

HORMONE	TARGET NEUROTRANSMISSIONS AND CEREBRAL AREAS	EFFECT
**Kisspeptin** (44, 45, 47, 48, 72)	Hypothalamus, amygdala, hippocampus, habenula, periaqueductal gray, ventral tegmental area (Animal and human studies) α2-adrenergic and serotoninergic neurotransmission (Animal studies)	Attenuation of negative mood, enhancement of the activity of prefrontal cortex in response to negative stimuli and of limbic areas in response to sexual stimuli (Human studies)
**GnRH** (49, 50)		Antidepressant and anxiolytic effect (Animal studies)
**Androgens** (51–55, 63–65, 69, 70)	Hypothalamus, amygdala, caudate, putamen, nucleus accumbens, preoptic area, lateral ventral septum, stria terminalis (Animal studies) Dopaminergic neurotransmission (Animal studies)	Neuroprotective effect Synaptic density in the hippocampus (Animal studies) Grey matter volume in hypothalamus, amygdala, hippocampus, mammillary bodies Activation of hippocampus and amygdala in response to fearful stimuli (Human studies)
**Estrogens** (56–60)	Hypothalamus, amygdala (ER α) Hippocampus, temporal cortex, claustrum, thalamus (ER β) (Human studies) Serotoninergic neurotransmission (Animal studies)	Antidepressant and anxiolytic effect (Animal studies)

GnRH, gonadotropin releasing hormone; ER, estrogen receptor.

However, the modulation of mood, as well as that of HPT axis functioning, are highly complex and integrated. Many other factors contribute to the tuning of these two systems, so that the reciprocal influence is hard to be clearly quantified. Observational and intervention studies can help define the clinical relevance of either alteration, and are summarized below.

## 4 Association between male sexual health and depression

### 4.1 Plasma sex steroid levels in depression

As stated above, depressive disorders can be associated with HPT axis dysfunction, however research into the relationship between plasma testosterone levels and major depression is complicated by etiologic and diagnostic uncertainties (74).

Three important epidemiological studies assessed the association between plasma testosterone levels and depressive symptoms, leading to controversial results: the Massachusetts Male Aging Study (MMAS) (75), the Veterans’ Experience Study (76) and the Rancho Bernardo Study (17). The MMAS was a population-based survey of 1709 men aged 40-70 years (75). Participants were required to fill a self-reported depression questionnaire, the Center for Epidemiologic Studies Depression Scale (CES-D), and to provide a morning blood sample for testosterone measurement. The statistical analysis showed no association between serum testosterone levels and CES-D-diagnosed depression. The Veterans’ Experience Study investigated a sample of 4393 veterans who served the U.S. military (mean age 37 years) (76). Participants were administered the Diagnostic Interview Schedule and provided morning blood samples for testosterone assessment. Plasma testosterone level was weakly but significantly correlated with depression, mania, and anxiety. Finally, in the Rancho Bernardo Study (17), 856 adult residents of a Californian community aged 50-89 years were enrolled in a 10-year follow-up study. They completed the Beck Depression Inventory and had a morning blood sample drawn for hormone assays. The study showed a significant inverse correlation between Beck Depression Inventory score and bioavailable, but not total, testosterone levels, pointing out that men with lower free testosterone levels had more severe depressive symptoms.

Other observational cross-sectional studies compared mean testosterone levels of depressed men with those of nondepressed controls (77, 78). Findings from such studies showed conflicting results (79). McIntyre et al. (80) assessed and compared total testosterone (TT) and bioavailable testosterone (BT) levels in two groups of middle-aged men (40-65 years): untreated subjects meeting DSM-4 criteria for a major depressive episode (N 44), and a matched nondepressed control group (N 50). Mean BT and TT levels were lower in the depressed group compared to the control one. Biochemical hypogonadism (i.e., BT level ≤ 70 ng/dL or TT level ≤ 350 ng/dL) was also more prevalent among depressed men than in nondepressed controls (34% versus 6%; 61% versus 14%, respectively). Giltay et al. (18) carried out a prospective 2-year study of testosterone levels in men with major depressive disorder as defined by the DSM-4 criteria compared with age matched healthy controls with mean age 70.5 ± 7.3 years. The study showed that the depressed group had lower mean testosterone levels than non-depressed controls after correction for age, level of education, body mass index, physical activity, smoking status, alcohol use, number of chronic diseases, and androgen-affecting medication. Of the 5.4% of subjects with total testosterone level lower than 230 ng/dL, 90% met criteria for major depressive disorder. These data underline the association between low testosterone levels and depression, demonstrating the presence of a graded risk of depression based on testosterone levels (81).

In contrast with the former considerations, however, other cross-sectional studies showed no difference in testosterone levels between depressed men and healthy controls (82–85).

Increased levels of estradiol were observed in depressed men in a case-control study by Fischer and colleagues (16). However, association of estradiol levels with depressive symptoms was confirmed in female, but not male, patients with HIV infection (86). Moreover, depressed mood showed no improvement after a short course of estradiol treatment in older men receiving androgen deprivation therapy (87).

In conclusion, the relationship between depression and sex steroids’ levels needs to be clarified yet. Evidence is limited and controversial, but seems to suggest an association, although weak, between low levels of testosterone and depressive symptoms in males (88).

### 4.2 Sexual symptoms in depression

According to the World Health Organization, sexual health is defined as a state of physical, emotional, mental, and social well-being related to sexuality. It is not merely an absence of disease, dysfunction, or infirmity, and it requires a positive and respectful approach to sexuality and sexual relationship (89). Typical manifestations of sexual dysfunction in men comprise erectile dysfunction, ejaculatory disorders, particularly premature ejaculation, low libido and orgasmic difficulty (90, 91). Moreover, erectile dysfunction is the main sexual problem associated with mental health and quality of life in males (92).

#### 4.2.1 Erectile dysfunction in depression

Several reports have suggested that erectile function is affected negatively by depression (91, 93, 94). Data obtained from the MMAS were revised in an analytic model by Araujo et al. (95) and showed that erectile dysfunction was associated with depressive symptoms, after controlling for potential confounders, with an odds ratio of 1.82. The authors concluded that the relationship between depressive symptoms and erectile dysfunction in middle-aged men is robust and independent of aging and para-aging confounders.

Takao and colleagues (96) looked at the prevalence of erectile dysfunction, assessed by the International Index of Erectile Function 5, among 87 Japanese patients with functional hypogonadism, 34 of which were diagnosed as having depression by the Mini International Neuropsychiatric Interview. They found that International Index of Erectile Function 5 scores of depressed patients were significantly lower than those of non-depressed patients.

In a multicenter study carried out in Brazil, Italy, Japan, and Malaysia, depression was shown to be associated with erectile dysfunction in a graded manner, and men with erectile dysfunction were 2.09 times more likely to have depression (97).

#### 4.2.2 Other sexual symptoms in depression

Many studies have documented an association between depression and sexual function. Howell et al. compared a population of depressed men by means of the Derogatis Sexual Functioning Inventory, a retrospective questionnaire on sexual function, with a group of age-matched healthy controls (98). They found that depressed men had lower sexual desire, a poorer self-image, and less sexual satisfaction, despite no significant difference in the frequency of sexual episodes.

Rizvi et al. carried out a study on 44 untreated depressed males and 50 age-matched healthy controls (78). Both populations had blood samples drawn to determine morning levels of total testosterone, sexual function outcomes measured using the Sex Effects Scale and depression severity assessed with the Hamilton Rating Scale for Depression-17 item. 27.9% of men were defined as hypogonadal and among these, men with major depressive disorder had lower scores on all domains of sexual function, especially orgasm and desire, compared to hypogonadal healthy controls. Multiple linear regression analyses revealed that depression status was the main factor influencing sexual function.

### 4.3 Depression in male hypogonadism

Some clinical manifestations of hypogonadism in males may overlap with those typical of major depression, like anxiety, insomnia, memory impairment and reduced cognitive function (88). However, it is not known what proportion of hypogonadal men meet criteria for major depression (74).

Korenman et al. conducted a retrospective study on 186 male hypogonadal men (mean testosterone values < 10.4 nmol/L) with mean age 18-40 years to test the hypothesis of a high prevalence of depression in young men with functional hypogonadism (99). They compared their demographic factors, other diagnoses and treatments with those of 3 different populations: 1) general population, 2) a population of 930 controls matched for age, BMI and alcohol use, 3) 404 controls with normal testosterone determinations and no hypogonadism diagnosis. Depression, defined as either an International Classification of Diseases, Ninth Revision (ICD-9) diagnosis, or treatment with an antidepressant medication, was found in 22.6% of cases vs 6.6% of the general population (P < 0.001). The matched controls had a depression rate of 13.4% (P < 0.002). Controls with normal testosterone determinations had a depression rate of 16.8% (P = 0.121).

Studies by Burris et al. (100), Wang et al. (101, 102), and Lašaite et al. (103) reported on mood in young men with functional hypogonadism compared to men with normal testosterone levels, showing a significant association between hypogonadal state and depressive symptoms.

Male patients undergoing androgen deprivation therapy for prostate cancer constitute an interesting clinical model to study the effects of testosterone deficiency. A greater increase in Beck Depression Inventory score has been observed in patients receiving androgen deprivation therapy compared to those treated with prostatectomy only (104). Variation in Beck Depression Inventory was proportional to severity of pre-existing erectile dysfunction, increase in body mass index and reduction of testosterone concentrations. A systematic review of clinical studies reporting on incidence of depression among individuals exposed to androgen deprivation therapy compared to non-exposed patients, found that androgen deprivation therapy conferred a 41% increased risk of depression (relative risk 1.41; 95% confidence interval 1.18–1.70; p<0.001) (105).

In conclusion, these association studies document a high incidence of depressive symptoms in male hypogonadism, and underline the importance of investigating these symptoms in young hypogonadal men.

### 4.4 Conclusions

The association between depression, testosterone levels and sexual symptoms in males is difficult to assess, due to numerous confounding factors, such as medical conditions, obesity, smoking, alcohol use, diet, and stress. Overall, current evidence shows that these conditions are linked and influence each other bidirectionally, with each factor reinforcing the other. However, additional studies are needed to investigate the relationship of testosterone levels with psychiatric symptoms.

Although there is not enough evidence at this time to recommend routine monitoring for plasma testosterone levels in patients with major depressive disorder, clinicians must be sensibilized on this topic and should keep in mind the potential link between these conditions.

## 5 Effects of antidepressants on HPT axis and sexual symptoms

### 5.1 Physiology of the sexual response

As described above, a mutual relationship between depression and sexual symptoms has been suggested by several studies. From this perspective, medications able to improve depressive disorders could also lead to an improvement in sexual symptoms. However, psychotropic drugs can negatively affect sexual function and behaviour. Eventually, knowledge of biology of the sexual response in humans is crucial to understand the potential effects of these drugs.

Sexual response includes 3 phases: libido or desire, arousal - resulting in erections in men and genital lubrication and swelling in women – and orgasm (106, 107). Many neurotransmitters (eg, dopamine, serotonin, acetylcholine, nitric oxide, noradrenaline) as well as hormones (prolactin, testosterone, oestrogen) are involved at various levels in this process (106–108).

The mesolimbic system plays a primary role in sexual motivation and dopamine is the most important neurotransmitter in this context. In fact, dopamine activation of the nucleus accumbens and medial preoptic hypothalamic region is fundamental for sexual interest (106). On the other hand, serotonin determines at this level an inhibitory effect on libido, in particular with involvement of the hippocampus and amygdala (109). It is noteworthy that central effects of serotoninergic system vary according to the activation of different receptor subtypes: in particular, receptors 5-HT_2_ and 5-HT_3_ have an inhibiting influence on sexual activity, whereas the activation of 5-HT_1_A stimulates sexual response.

Sexual arousal involves mesolimbic structures and peripheral autonomic sympathetic and parasympathetic nervous system. The entire process is influenced by several neurotransmitters, including dopamine, serotonin, acetylcholine and noradrenaline. Furthermore, nitric oxide, a mediator of vasodilation, also plays a relevant role in the erectile tissues (106, 109, 110).

Finally, orgasm and ejaculation are mediated through hypothalamus and peripheral sympathetic nervous system where serotonin, prolactin and noradrenaline contribute to the regulation of this phase (106).

### 5.2 Effects of psychotropic treatments: antidepressants

Antidepressant drugs can affect all 3 phases of normal sexual response in males by reducing libido, causing arousal disturbance or delaying orgasm. However, the rate and severity of sexual symptoms vary among antidepressants according to different central and autonomic actions (111).

First-generation antidepressant [tricyclic antidepressants (TCAs) and Monoamine oxidase inhibitors (MAOIs)] such as antidepressants with serotoninergic activity [selective serotonin reuptake inhibitors (SSRIs) and serotonin/norepinephrine reuptake inhibitors (SNRIs)] are frequently associated with sexual dysfunction (111–113).

A meta-analysis including double-blind, single-blind, open-label, cross-sectional and retrospective studies reported a rate of treatment-emergent sexual symptoms ranging from 25 to 80%, with the highest risk observed for SSRIs citalopram, fluoxetine, paroxetine, sertraline and for the SNRI venlafaxine (110).

First-generation antidepressants increase central availability of serotine and noradrenalin and exert an anticholinergic action. Serotoninergic activity in turn interferes with dopaminergic signal in the mesolimbic system and can also lead to an increase of prolactin levels with subsequent hypogonadotropic hypogonadism (114–116).

SSRIs and SNRIs can induce sexual dysfunction through multiple mechanisms. As described above, activation of 5-HT_2_ receptors results in the central inhibition of sexual circuits likely due to decreased dopaminergic transmission (110). Inhibition of peripheral autonomic sympathetic and parasympathetic nervous systems can also contribute to the onset of sexual side effects. Finally, SSRIs can decrease production of nitric oxide by interfering with nitric oxide synthetase (111, 113, 117). Among SNRIs, the balance between the degree of serotonin and norepinephrine reuptake inhibition changes the impact of these drugs on sexual function (namely, the greater the inhibition of serotonin reuptake, the greater the incidence of sexual effects). Accordingly, in their meta-analysis Serretti and coll. found that venlafaxine, a potent serotonin reuptake inhibitor, is the SNRI with the highest risk of dysfunction (110).

Conversely, other antidepressants such as agomelatine (an analogue of melatonin which combines 5HT_2_C antagonism with melatonin receptors 1 and 2 agonism) (118), amineptine and bupropion (both dopamine reuptake inhibitors), nefazodone (a 5-HT_2_ receptor antagonist) and mirtazapine (an antagonist of central α_2_-adrenergic, 5-HT_2_ and 5-HT_3_ receptors) did not show a higher risk with respect to placebo (110).

Another meta-analysis including data from 63 studies (58 randomized controlled trials, 5 observational studies) with more than 26,000 patients confirmed that second-generation antidepressants are related to an increased risk of sexual dysfunction. Furthermore, male-specific analysis showed the highest risk with sertraline and paroxetine. Conversely, bupropion showed a statistically significantly lower risk of dysfunction than other second-generation antidepressants (119).

In a recent systematic review and meta-analysis considering only randomized controlled studies, Trinchieri and coll. evaluated the effect of second-generation antidepressants on the male sex cycle (113). The analysis included 22 studies and showed an overall increased odds ratio (OR) for decreased libido (OR=1.89), erectile dysfunction (OR=2.28) and ejaculatory dysfunction (OR=7.31) in patients treated with antidepressants compared with placebo, thus confirming the results of previous studies.

Interestingly, subgroup analysis for SSRIs and SNRIs showed that both classes of antidepressants were associated with higher OR of decreased libido and ejaculatory dysfunction with respect to placebo. Conversely, SNRIs but not SSRIs presented higher OR of erectile dysfunction, suggesting a specific effect of SNRIs on this phase of sexual cycle possibly related to dose-dependent noradrenaline-mediated vasoconstriction at peripheral sympathetic level (113).

Very recently, Winter and coll. performed a systematic literature search, including case studies, case reports, and clinical trials with the aim to assess the sexual impact of antidepressant treatments in patients with major depressive disorders (116). In addition to confirming the association between sexual dysfunction and therapy with SSRIs, SNRIs, TCAs and MAOI phenelzine, the study focused on some antidepressants having a more favourable profile on sexual function. Among these, vortioxetine (a 5-HT_3_, 5-HT_7_ and 5-HT_1_D receptor antagonist, 5-HT_1_B receptor partial agonist, 5-HT_1_A receptor agonist and serotonin transporter inhibitor) and vilazodone (a serotonin transporter inhibitor, 5-HT_1_A partial agonist with low affinity for 5-HT_1_D, 5-HT_2_A and 5-HT_2_C receptors), both approved for the treatment of major depressive disorders, were characterized by a low risk of sexual dysfunction, in particular at low doses. Finally, as previously described, the prevalence of sexual disorders in patients treated with bupropion and mirtazapine was similar to placebo (116).

### 5.3 Effects of psychotropic treatments: atypical antipsychotics

Atypical (or second-generation) antipsychotics (AAPs) are frequently prescribed as augmenting agents in patients with major depression in case of incomplete clinical response to monotherapy with antidepressants (116). Similarly to the latter, also AAPs can induce sexual disorders - in particular erectile dysfunction, ejaculation disorders and reduced intensity of orgasm - with an overall prevalence rate of 54% (109). AAPs-induced sexual dysfunction is mainly related to the antagonism of these drugs for D2 and 5-HT_2_A receptors in the brain. In addition to harming the previously described direct central effects of dopamine in the mesolimbic area, blocking D2 receptors in the tuberoinfundibular pathway also increases prolactin levels, thus leading to an increase of opioid and GABA levels as well as a reduction of testosterone levels resulting in hypogonadotropic hypogonadism. Other possible mechanisms of action of AAPs include central alpha-adrenergic, anticholinergic and antihistaminergic effects, which can induce sedation and reduce peripheral vasodilatation (109, 113). Of note, the peripheral effect on adrenergic system, through blocking of apha1-adrenergic receptors, account for the onset of priapism, another sexual dysfunction frequently reported during treatment with these drugs, especially olanzapine and risperidone (120, 121). Meta-analytic studies showed that among AAPs, those most affecting prolactin secretion, such as risperidone, olanzapine and clozapine were associated with a higher risk of sexual side effects (40-60%), whereas quetiapine, aripiprazole, ziprasidone and perphenazine were associated with a lower risk (16-27%) (122, 123). In particular, risperidone (characterized by a high affinity for D2, α_1_- and α_2_-adrenergic receptors) and aripiprazole (partial D2 agonist with lower affinity for α_1_-adrenergic receptors) seem to be AAPs with greater and lesser risk of sexual dysfunction, respectively (113, 124).

**Figure 1:**
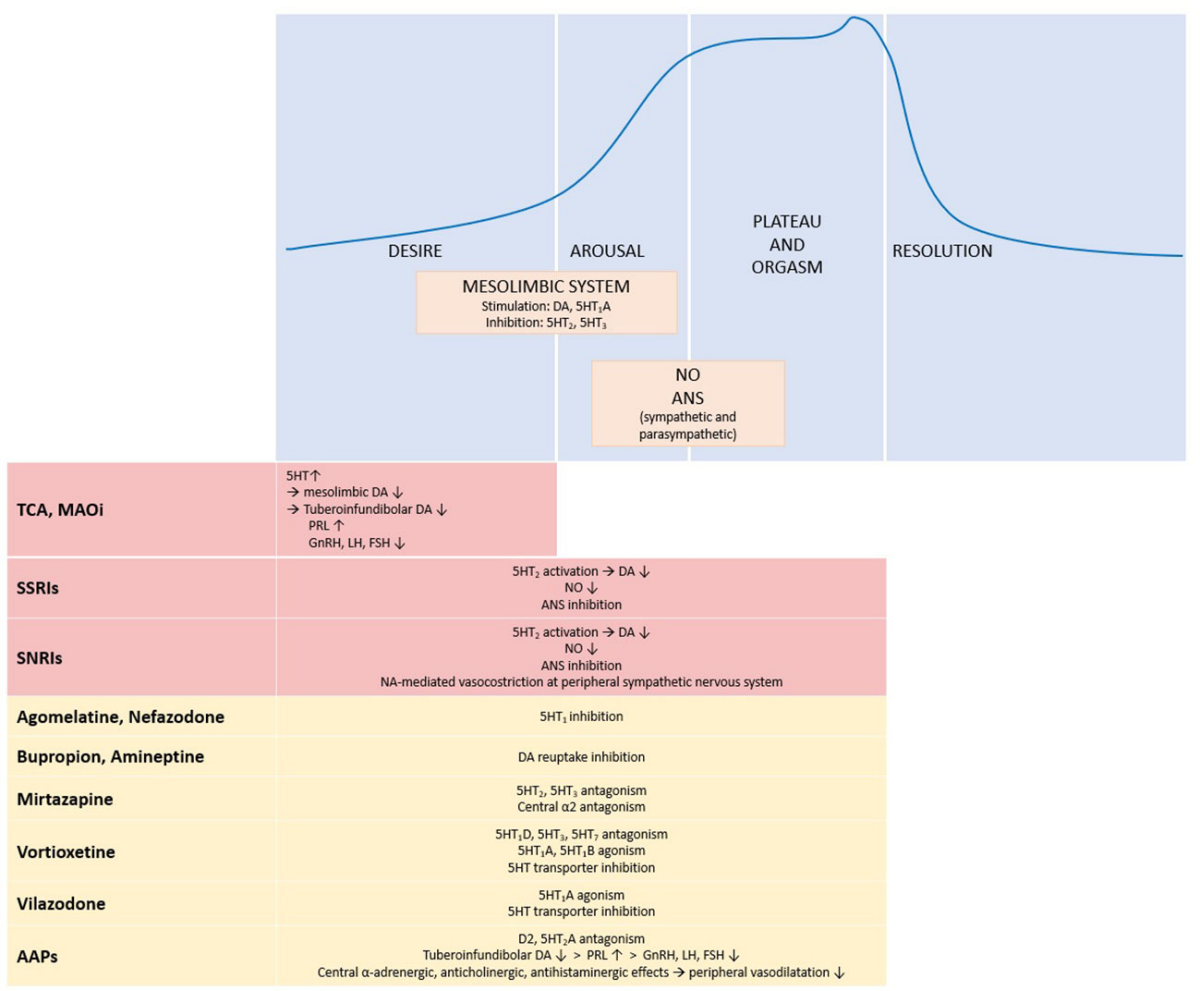
Physiology of sexual response and effects of antidepressant and antipsychotic drugs. The upper panel shows the four phases of sexual response and the main systems and neurotransmitters involved. The lower panel summarizes the effects of antidepressant and antipsychotic drugs on the different phases of the sexual response cycle: red boxes stand for inhibitory effects, yellow boxes for neutral effect. ↑, increase; ↓, decrease; 5HT, serotonin; 5HT1, serotonin receptor 1; 5HT_1_A, serotonin receptor 1A; 5HT_1_B, serotonin receptor 1B; 5HT_1_D, serotonin receptor 1D; 5HT_2_, serotonin receptor 2; 5HT_3_, serotonin receptor 3; 5HT_7_, serotonin receptor 7; α2, α2 adrenergic receptor; AAPs, atypical antipsychotics; ANS, autonomic nervous system; DA, dopamine; D2, dopamine receptor 2; FSH, follicle stimulating hormone; GnRH, gonadotropin releasing hormone; LH, luteinizing hormone; MAOi, monoamine oxidase inhibitors; NA, norepinephrine; NO, nitric oxide; PRL, prolactin; SNRIs, serotonin/norepinephrine reuptake inhibitors; SSRI, selective serotonin reuptake inhibitors; TCA, tricyclic antidepressants.

**Figure 1** summarizes the effects of antidepressant and antipsychotic drugs on sexual response.

## 6 Effects of testosterone replacement therapy on depression

### 6.1 Effects of TRT on depression in mixed populations (hypogonadal and eugonadal subjects)

To date, only few placebo-controlled randomized clinical trials (RCTs) aimed to describe the effect of TRT on depressive symptoms have been performed.

In 2009 Zarrouf and coll. published a meta-analysis including 7 RCTs evaluating androgen replacement therapy in different patient populations with DSM-4-defined depressive disorders, ranging from dysthymia/minor depression to major depressive disorder (125). In particular, four trials used intramuscular testosterone therapy, one trial oral dehydroepiandrosterone (DHEA) and two testosterone gel. Moreover, 5 studies included patients with low levels of total testosterone, while 2 evaluated eugonadal subjects. Overall, meta-analysis showed a significant positive impact of androgen therapy on Hamilton Rating Scale for Depression response with respect to placebo. A subgroup analysis confirmed the beneficial effect of both TRT and DHEA on depressive symptoms, while no significant changes in eugonadal subjects were found (125).

A subsequent meta-analysis of Amanatkar and coll. selected 16 RCTs (944 patients) including subjects with and without hypogonadism evaluated for mood disorders through mixed questionnaires (126). Nine trials were performed in hypogonadal patients, 3 trials in subjects with normal testosterone levels, and 4 in both populations. Similarly to the aforementioned work, this meta-analysis also showed in the whole population a positive effect of testosterone on mood compared with placebo. Interestingly, subgroup analysis found significant effects in men younger than 60 years, in hypogonadal patients and in the case of subthreshold depression. On the contrary, testosterone therapy did not result effective in men older than 60 years, in eugonadal population and in patients with major depressive disorder (126).

Finally, in 2018 Walther and coll. summarized in their meta-analysis the results of 27 RCTs (1890 patients) including heterogenous populations with diverse medical conditions and mood disorders (127). While this study confirmed a moderate positive effect of testosterone treatment on depression compared with placebo, it also showed, in contrast to previous work, that initial testosterone status as well as age were not moderators of the effect of testosterone treatment on depressive symptoms.

### 6.2 Effects of TRT in hypogonadal patients with pre-treatment mild depression or major depressive disorder

In a setting where only patients with hypogonadism were considered, Elliott and coll. evaluated the effect of TRT on many outcomes, including quality of life, depression, sexual function, metabolic and adverse events (128). As far as depressive symptoms are concerned, the analysis of 12 RCTs (852 patients) showed that, compared with placebo, treatment with any TRT improved depression. However, no subgroup analysis based on different depressive disorders was carried out.

More recently, Vartolomei and coll. performed a systematic review with the aim to analyze the effect of TRT on depression and depressive symptoms in adult patients affected by late-onset hypogonadism (129). For the analysis, RCTs including at least 20 men with age >30 years and total testosterone levels <350 ng/ml, treated with TRT compared with placebo, were considered. Moreover, the Authors studied the impact of TRT on well-established pre-treatment depressive disorders as primary outcome, and its impact in patients without pre-treatment depression as secondary outcome. Similarly to the meta-analysis described above, TRT showed a positive impact on mood in patients with late-onset hypogonadism with clinical mild depression diagnosed before treatment. Moreover, TRT did not show a significant effect within the complex spectrum of major depressive disorder, in which TRT should probably be included in a multimodal therapeutic approach.

### 6.3 Effects of TRT in hypogonadal patients without pre-treatment depression

Finally, the same systematic review analysed the role of TRT in patients suffering from late-onset hypogonadism without clinically significant depression before treatment. Despite conflicting results between studies, the overall analysis suggested a beneficial effect of TRT compared with placebo, resulting in a reduction of depressive symptoms. However, clinical implication of this effect must be clarified as well (129).

### 6.4 Conclusions

The effects of TRT on depression have been investigated in few and relatively small-sized RCTs that were heterogeneous for study populations, testosterone doses and formulation, endocrinological and psychiatric eligibility criteria, intervention durations, and outcome ascertainment. Overall, they found TRT beneficial, with significant improvement among those patients with hypogonadism and clinical mild depression. On the other hand, in patients affected by major depression testosterone therapy seems to be ineffective. It is also important to consider that major depressive disorder is a complex disease, in which the neurobiological role of testosterone as well as the safety of TRT need to be further clarified, also considering previous suggestions on the relationship between testosterone levels and the course of bipolar disorders and suicidal behaviour (130).

Taking into account these evidences, recent guidelines recommended against the use of TRT with the sole purpose of improving major depressive symptoms in subjects with hypogonadism (1).

## 7 Conclusions

Several observations suggest a bidirectional influence between male hypogonadism and mood regulation, with each factor reinforcing the other. Epidemiological and observational studies highlight that men suffering from depression have lower circulating testosterone levels (17, 75, 76) and higher prevalence and greater severity of sexual symptoms (78, 95, 96, 98). On the other hand, men with hypogonadism can manifest depressive symptoms (6–10) or even receive a diagnosis of major depression (99) more frequently than eugonadal controls. However, the prevalence of functional hypogonadism in major depression, and that of major depression among hypogonadal men, are not currently known, and some studies have not confirmed this association (11, 82–85). It is noteworthy that available studies are largely heterogeneous with regard to population included (age, comorbidities, alcohol or substance abuse), definition of depression (questionnaires, DSM, ICD-9, prescription of antidepressants) and of male hypogonadism (total or bioavailable testosterone concentrations, and/or sexual dysfunction). Furthermore, if depression is a cause of functional hypogonadism, an improvement of sexual symptoms would be expected when patients are treated with anti-depressant medications. However, most psychotropic drugs are known to negatively affect sexual function and behaviour because of their specific mechanism of action.

Under a mechanistic perspective, several cerebral areas, involved in depression physiopathology, express the receptors and respond to HPT axis hormones (45, 54, 57). *In vivo* studies have shown that circulating levels of sex hormones correlate with grey matter volume, synaptic density (63–65), and functioning of critical regions (66–71). Moreover, animal and human studies have shown that administration of exogenous kisspeptin or GnRH can influence adrenergic and serotoninergic transmission, modulate activity of limbic areas and prefrontal cortex, and exert antidepressant effects (48–50, 72). However, the same areas directly receive neuronal fibers containing kisspeptin and GnIH/RFRP (44), express enzymes which metabolize sex hormones (e.g. aromatase) (56), and may even synthetize steroid hormones locally (so-called neurosteroids) (61). Therefore, the effect of HPT axis hormones on these cerebral circuits may add to local regulation. Moreover, other biological aspects, like polymorphisms of androgen receptor, may further complicate the relationship between circulating levels of sexual hormones and their actual effects on the nervous system.

Overall, some clinical suggestions emerge from available literature. Firstly, sexual dysfunction should be investigated in men suffering from depression. If sexual symptoms are present and reported as relevant to the patient’s quality of life, testosterone levels should be assessed. If a diagnosis of male hypogonadism is established (1–3), we suggest that TRT may be offered to the patient, and other sexual symptoms (e.g. erectile dysfunction) should be managed properly. If sexual complaints develop while on antidepressant treatment, switching to antidepressant or antipsychotic medications with lower risk of sexual dysfunction, could be considered (i.e. bupropion, mirtazapine, vortioxetine, vilazodone, aripiprazole) (116, 124). Conversely, the use of TRT with the sole purpose of improving depressive symptoms is not recommended according to current evidence (1). TRT should be periodically reassessed as, along with resolution of the depressive episode, testosterone discontinuation may be attempted. Moreover, in patients with bipolar disorder, testosterone therapy should be carefully weighed, as we hypothesize that testosterone treatment may increase the risk of manic episodes and suicide attempts (130), and presumably worsen hypersexuality symptoms.

On the other hand, mood disorders, anxiety, insomnia, memory impairment and reduced cognitive function should be evaluated in men diagnosed with hypogonadism before and after starting TRT, in order to assess the response of these symptoms to treatment, and to identify those conditions which deserve a specialist consultation.

Finally, larger longitudinal studies are needed to document changes in HPT axis functioning and sexual symptoms in men during depressive episodes and remission phases, to explore correlation of male hypogonadism with some physiopathological aspects like sleep disruption, hypothalamus-pituitary-adrenal axis hyperactivity, weight loss and psychomotor retardation, and, lastly, to better understand the clinical relevance of male hypogonadism in major depression.

## Author contributions

RI, VL and EF conducted the literature search and wrote the manuscript. MA and GM critically revised the manuscript. All authors contributed to the article and approved the submitted version.
